# Data for comparative proteomics analysis of the antitumor effect of CIGB-552 peptide in HT-29 colon adenocarcinoma cells

**DOI:** 10.1016/j.dib.2015.06.024

**Published:** 2015-07-08

**Authors:** Teresa Núñez de Villavicencio-Díaz, Yassel Ramos Gómez, Brizaida Oliva Argüelles, Julio R. Fernández Masso, Arielis Rodríguez-Ulloa, Yiliam Cruz García, Osmany Guirola-Cruz, Yasset Perez-Riverol, Luis Javier González, Inés Tiscornia, Sabina Victoria, Mariela Bollati-Fogolín, Vladimir Besada Pérez, Maribel Guerra Vallespi

**Affiliations:** a Department of Systems Biology, Center for Genetic Engineering and Biotechnology, Cuba; b Pharmaceuticals Department, Center for Genetic Engineering and Biotechnology, Cuba; c Department of Preclinical Studies, National Institute of Oncology and Radiobiology of Cuba, Cuba; d European Molecular Biology Laboratory, European Bioinformatics Institute (EMBL-EBI), Wellcome Trust Genome Campus, Hinxton, Cambridge, UK; e Cell Biology Unit, Institut Pasteur of Montevideo, Uruguay

**Keywords:** Colorectal cancer, Apoptosis, Inflammation, CIGB-552 synthetic peptide, Enrichment analysis, Text mining

## Abstract

CIGB-552 is a second generation antitumor peptide that displays potent cytotoxicity in lung and colon cancer cells. The nuclear subproteome of HT-29 colon adenocarcinoma cells treated with CIGB-552 peptide was identified and analyzed [1]. This data article provides supporting evidence for the above analysis.

**Table:** Specifications Table

Subject area	Biology
More specific subject area	Pharmacology, Proteomics
Type of data	Figure, table, methods
How data was acquired	Mass spectrometry: hybrid quadrupole orthogonal acceleration tandem mass spectrometer QTof-2 (Micromass, Manchester, U.K.)
Data format	Analyzed
Experimental factors	Isolation of the Nuclear Proteins Enriched Fraction, trypsin digestion and isotope labeling of peptides
Experimental features	Subcellular fractionation, protein and peptide fractionation by DF-PAGE and LC-MS/MS peptide identification
Data source location	Havana, Cuba
Data accessibility	The data are provided in this article.

## Value of the data

• The data details the DF-PAGE separation method used in the proteomics analysis of GIGB-552 peptide effect.
• The data details the bioinformatics-driven approach used for the functional classification of the identified and differentially modulated proteins.
• The data provides an overview of the nuclear proteins differentially modulated in HT-29 colon adenocarcinoma cells treated with the antitumor peptide CIGB-552 and their functional classification.

## 1 Data, experimental design, materials and methods

We performed a comparative proteomics experiment in duplicate focusing on the quantification of the nuclear subproteome of the human HT-29 colon adenocarcinoma cells via a dual-fractionation by polyacrylamide gel electrophoresis (DF-PAGE) approach. The differentially modulated proteins were functionally analyzed using a systems biology workflow that integrates the information obtained from two main groups of bioinformatics tools [1].

## 2 Dual fractionation by polyacrylamide gel electrophoresis (DF-PAGE)

**Fig. 1:**
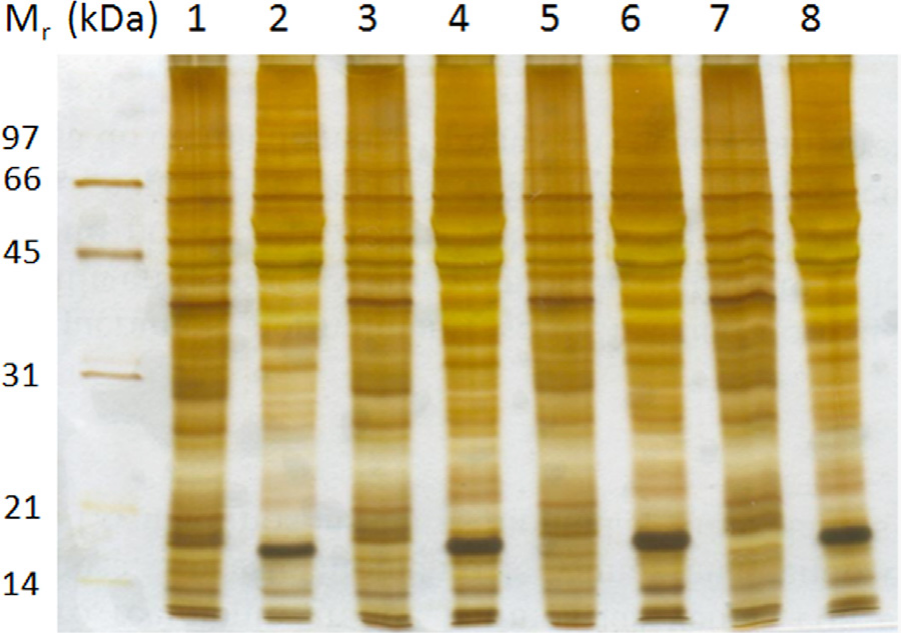
SDS-PAGE analysis of nuclear and PBS-Triton X-100 soluble fractions for both control and CIGB552-treated samples. Lanes 1,2: PBS-Triton X-100 soluble and nuclear fractions of control sample, experiment#1; Lanes 3,4: PBS-Triton X-100 soluble and nuclear fractions of CIGB552 peptide-treated, experiment#1; Lanes 5,6: PBS-Triton X-100 soluble and nuclear fractions of control sample, experiment#2; Lanes 7,8: PBS-Triton X-100 soluble and nuclear fractions of CIGB552 peptide-treated, experiment#2. The 12.5% T gel was silver stained according to standard procedures [12].

**Fig. 2:**
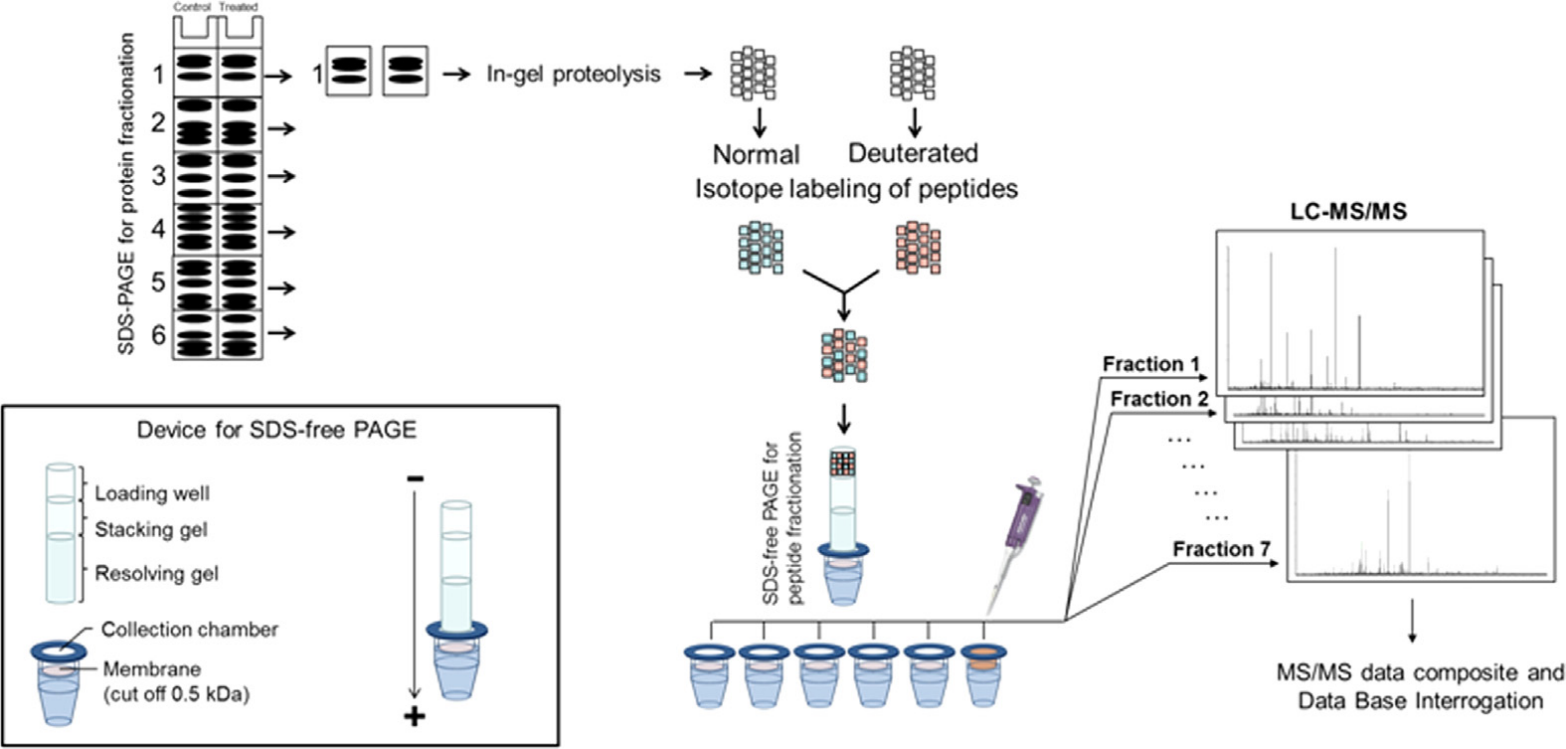
Schematic representation of DF-PAGE method for quantitative proteomics. Nuclear proteins from both, control and CIGB-552 peptide treated cells were fractionated by SDS-PAGE. Homologous fractions are in-gel digested with trypsin and further isotopically labeled with normal or deuterated N-acetoxy-succinimide. Samples from control and CIGB-552 peptide treated cells are mixed and fractionated by SDS-free PAGE. Collected fractions are then analyzed by LC-MS/MS.

**Fig. 3:**
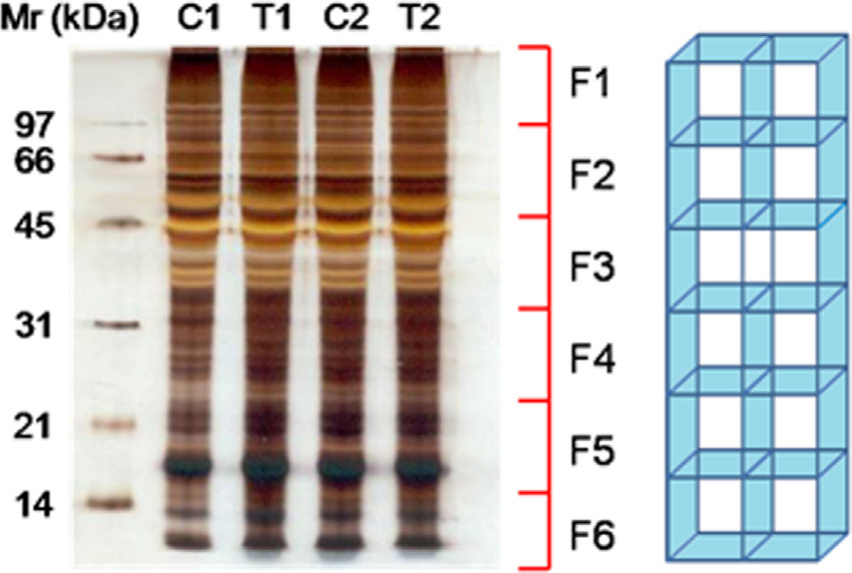
SDS-PAGE analysis of nuclear fractions obtained from control and CIGB-552 peptide treated cells of two independent experiments. At the right side is shown a schematic representation of the device used for cutting the gel and obtaining the SDS-PAGE fractions. The 12.5% T gel was silver stained according to standard procedures [12]. C1, T1: control and CIGB-552 peptide treated samples from the experiment#1. C2, T2: control and CIGB-552 peptide treated samples from the experiment#2. The number and protein mass range of the fractions F1-6 are indicated at the right side of the figure.

The DF-PAGE method combines sequentially, protein fractionation by SDS-PAGE, in-gel tryptic digestion and peptide fractionation by SDS-free PAGE [2,3]. In the first fractionation step (SDS-PAGE), proteins are solubilized in a SDS containing solution and separated according to their size. The presence of SDS ensures the solubilization of virtually all the proteins including highly hydrophobic proteins [2]. After the in-gel tryptic digestion, the peptide mixture is transferred to a second SDS-free gel. In the absence of SDS, peptides migrate according to their charge and size which is orthogonal to the peptide separation in RP-C18 during the LC-MS/MS analysis [2]. For quantitative DF-PAGE, isotope labeling of peptides is introduced with normal- or deuterated N-acetoxysuccinimide just before peptide fractionation by SDS-free PAGE. Fig. 1 shows a SDS-PAGE analysis of soluble and nuclear fractions for CIGB-552 peptide treated and control samples of two independent experiments. Fig. 2 represents a schematic representation of the DF-PAGE method [3]. Finally, Fig. 3 shows same data as Fig. 1 but for the nuclear fractions obtained from control and treated samples.

## 3 Bioinformatics-driven functional interpretation

**Fig. 4:**
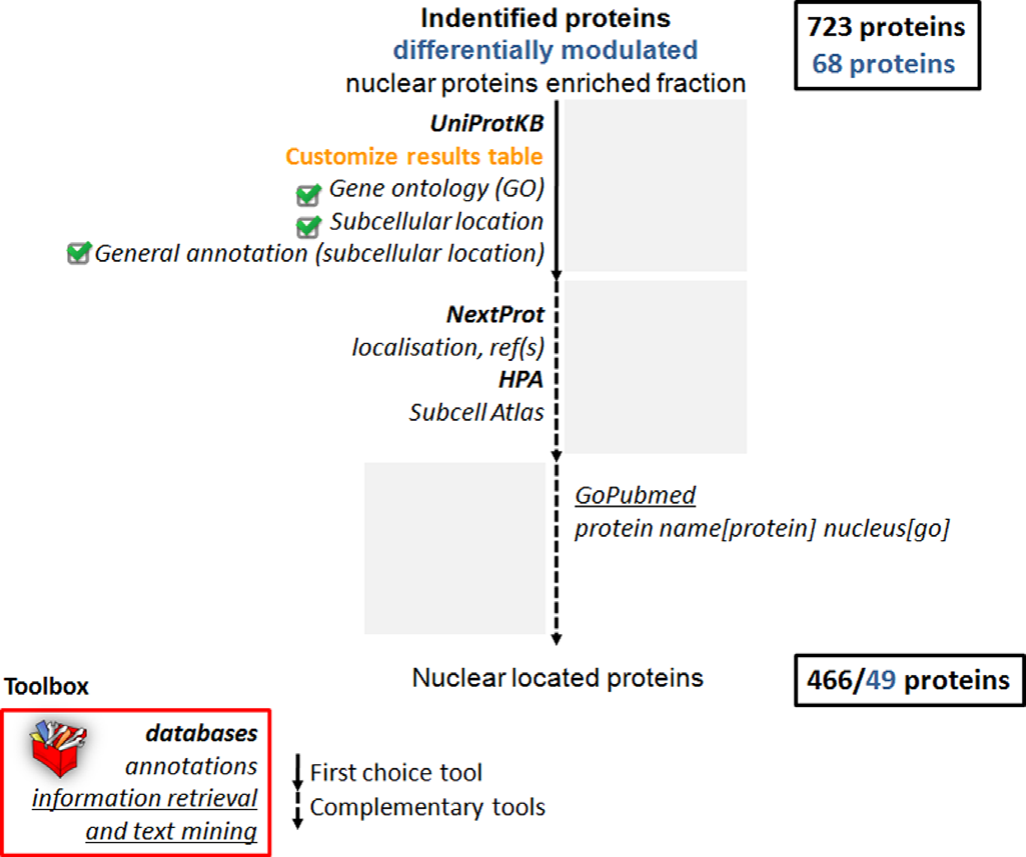
Schematic representation of the subcellular location classification procedure that shows the number of identified/differentially modulated proteins classified as nuclear located.

The functional interpretation of the comparative proteomics data was performed based on two main groups of bioinformatics tools: enrichment analysis and information retrieval and text mining [4]. Since we studied the nuclear subproteome, the identified proteins (Supplementary Table 1) were classified according to subcellular location using information from UniProtKB [5], NextProt [6] and HPA [7] databases and the literature (Supplementary Table 2). Fig. 4 summarizes the procedure used for nuclear protein classification and the results obtained. We considered UniProtKB as the primary resource and searched the literature and/or NextProt and HPA databases when information of nuclear localization was missing in the former. Literature information was obtained using GoPubMed [8] text mining tool defining the query as the protein name and nucleus as a GO annotation (Fig. 3).

**Fig. 5:**
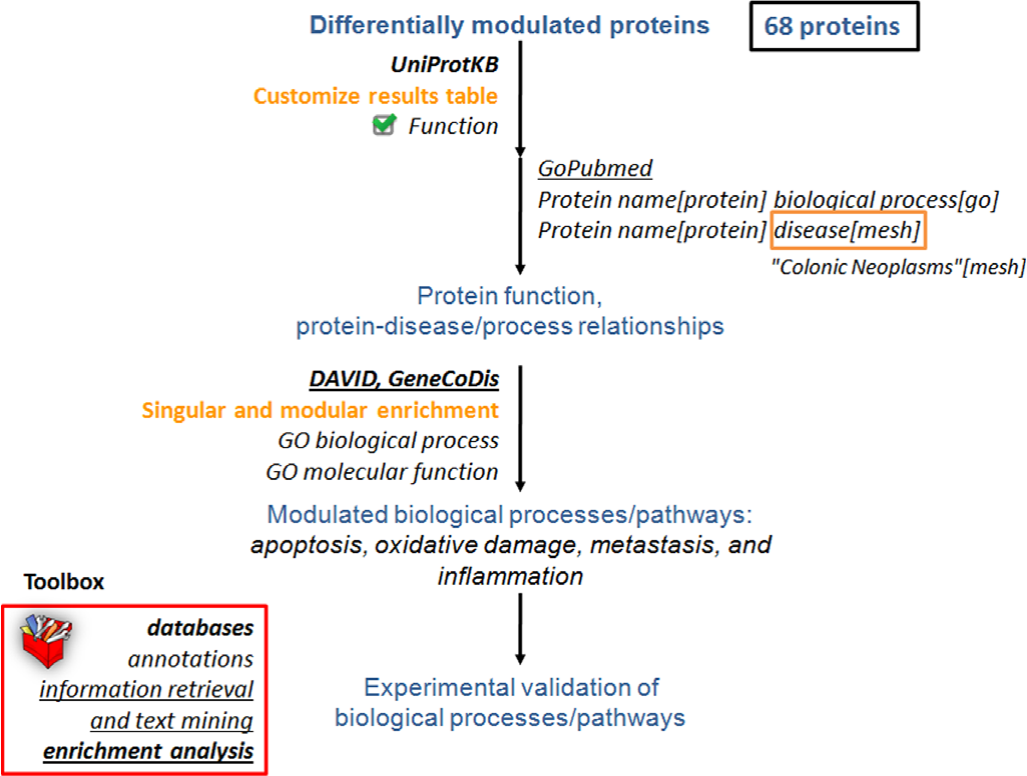
Schematic representation of the bioinformatics-driven functional interpretation procedure to study the function of the differentially modulated proteins and the results obtained which derives in the experimental validation of specific biological processes and pathways.

**Fig. 6:**
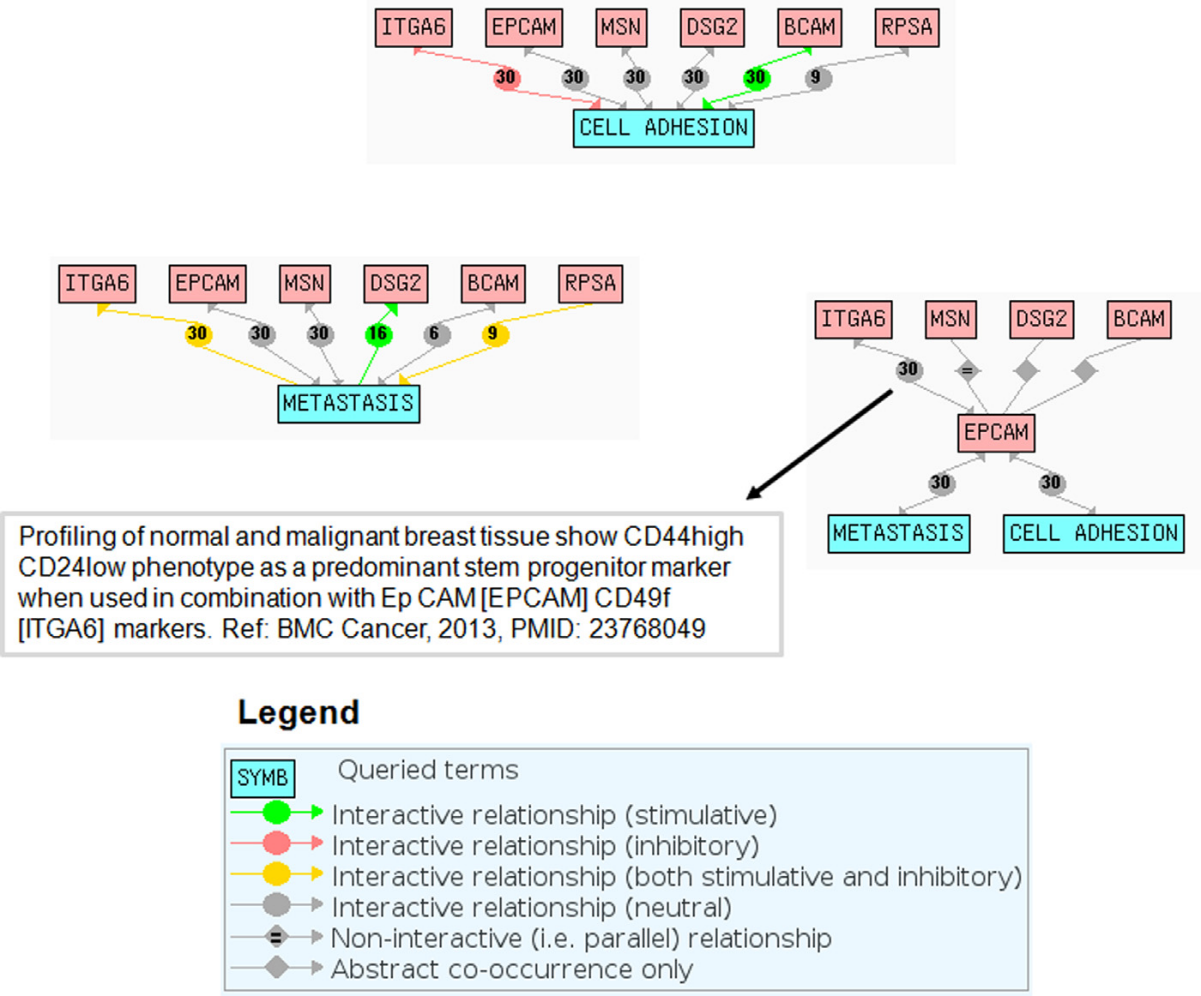
Chilibot text mining analysis for identifying functional relationships between a set of differentially modulated proteins and between these proteins and cell adhesion and metastasis.

The differentially modulated proteins were functionally analyzed using function information extracted from UniProtKB, GO biological process and molecular function enrichment analyses (Supplementary Table 3) and the literature. Fig. 5 shows a schematic representation of the followed procedure. Protein function and literature information on protein-disease/biological process relationships helped us to understand the relevance of the differentially modulated proteins. This information was combined with the results of the enrichment with DAVID [9] and GeneCoDis [10] tools to study the proteins not covered by this type of analysis. In addition, Fig. 6 illustrates the use of Chilibot [11] text mining tool to represent in a biological network format protein-biological process and protein–protein functional relationships.

## References

[1] Núñez de Villavicencio-Díaz T., Ramos Gómez Y., Oliva Argüelles B., Fernández Masso J.R., Rodríguez-Ulloa A., Cruz García Y. (2015). Comparative proteomics analysis of the antitumor effect of CIGB-552 peptide in HT-29 colon adenocarcinoma cells. J. Prot..

[2] Ramos Y., Gutierrez E., Machado Y., Sánchez A., Castellanos-Serra L., González L.J. (2008). Proteomics based on peptide fractionation by SDS-free PAGE. J. Prot. Res..

[3] Ramos Y., Besada V., Castellanos-Serra L. (2012). Peptide fractionation by SDS-free polyacrylamide gel electrophoresis for proteomic analysis via DF-PAGE. Methods Mol. Biol..

[4] Villavicencio-Diaz T.N., Rodriguez-Ulloa A., Guirola-Cruz O., Perez-Riverol Y. (2014). Bioinformatics tools for the functional interpretation of quantitative proteomics results. Curr. Top. Med. Chem..

[5] Boutet E., Lieberherr D., Tognolli M., Schneider M., Bairoch A. (2007). UniProtKB/Swiss-Prot. Methods Mol. Biol..

[6] Gaudet P., Argoud-Puy G., Cusin I., Duek P., Evalet O., Gateau A. (2013). NeXtProt: organizing protein knowledge in the context of human proteome projects. J. Prot. Res..

[7] Pontén F., Schwenk J.M., Asplund A., Edqvist P.-HD (2011). The Human Protein Atlas as a proteomic resource for biomarker discovery. J. Intern. Med..

[8] Doms A., Schroeder M. (2005). GoPubMed: exploring PubMed with the gene ontology. Nucleic Acids Res..

[9] Huang D.W., Sherman B.T., Lempicki R.A. (2009). Systematic and integrative analysis of large gene lists using DAVID bioinformatics resources. Nat. Prot..

[10] Tabas-Madrid D., Nogales-Cadenas R., Pascual-Montano A. (2012). GeneCodis3: a non-redundant and modular enrichment analysis tool for functional genomics. Nucleic Acids Res..

[11] Chen H., Sharp B.M. (2004). Content-rich biological network constructed by mining PubMed abstracts. BMC Bioinform..

[12] Heukeshoven J., Dernick R. (1988). Improved silver staining procedure for fast staining in phastsystem development unit. I. Staining of sodium dodecyl sulfate gels. Electrophoresis.

